# Implications of Oxidative Stress and Cellular Senescence in Age-Related Thymus Involution

**DOI:** 10.1155/2020/7986071

**Published:** 2020-02-05

**Authors:** Alexandra Barbouti, Panagiotis V. S. Vasileiou, Konstantinos Evangelou, Konstantinos G. Vlasis, Alexandra Papoudou-Bai, Vassilis G. Gorgoulis, Panagiotis Kanavaros

**Affiliations:** ^1^Department of Anatomy-Histology-Embryology, Medical School, University of Ioannina, Ioannina, Greece; ^2^Molecular Carcinogenesis Group, Department of Histology and Embryology, Medical School, National and Kapodistrian University of Athens, Athens, Greece; ^3^Department of Anatomy, Medical School, National and Kapodistrian University of Athens, Athens, Greece; ^4^Department of Pathology, Medical School, University of Ioannina, Ioannina, Greece; ^5^Faculty Institute for Cancer Sciences, Manchester Academic Health Sciences Centre, University of Manchester, Manchester, UK; ^6^Biomedical Research Foundation, Academy of Athens, Athens, Greece

## Abstract

The human thymus is a primary lymphoepithelial organ which supports the production of self-tolerant T cells with competent and regulatory functions. Paradoxically, despite the crucial role that it exerts in T cell-mediated immunity and prevention of systemic autoimmunity, the thymus is the first organ of the body that exhibits age-associated degeneration/regression, termed “thymic involution.” A hallmark of this early phenomenon is a progressive decline of thymic mass as well as a decreased output of naïve T cells, thus resulting in impaired immune response. Importantly, thymic involution has been recently linked with cellular senescence which is a stress response induced by various stimuli. Accumulation of senescent cells in tissues has been implicated in aging and a plethora of age-related diseases. In addition, several lines of evidence indicate that oxidative stress, a well-established trigger of senescence, is also involved in thymic involution, thus highlighting a possible interplay between oxidative stress, senescence, and thymic involution.

## 1. Introduction

The thymus is a central lymphoepithelial organ of the immune system. Its primary function is to provide a unique microenvironment in which T cell precursors (thymocytes), derived from hematopoietic stem cells, migrate and undergo selection, activation, clonal expansion, and differentiation into self-tolerant, immunocompetent T cells that are released to the periphery [1, 2]. Proper T cell development requires the interaction of thymocytes with critical cellular populations of the cortical and/or the medullary regions of the thymus, especially thymic epithelial cells (TECs) and dendritic cells (DCs) [1, 3–6], which regulate thymopoiesis through cell to cell contacts and production of soluble factors (e.g., chemokines, cytokines, and extracellular matrix components) [2, 7–11].

**Figure 1:**
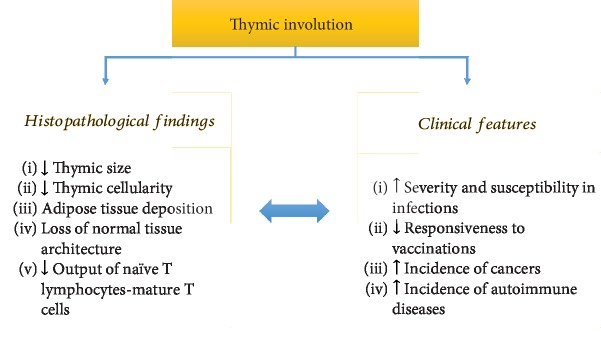
Summary of key histopathological findings and clinical manifestations of thymic involution.

Despite the fundamental requirement for lifelong establishment and maintenance of an overall effective and adequate defense against pathogens, the function of the immune system deteriorates with age, affecting both innate and adaptive immune responses (*immunosenescence*) [12, 13]. Surprisingly, the thymus is the first organ of the body that exhibits age-associated changes, known as thymus regression or involution, a biological event that takes place in almost all vertebrates, suggesting that this is an evolutionary ancient and conserved process [14, 15]. Although normal (disease-free) aging has a significant effect in the mass of specific high-metabolic-rate organs (for example, the brain, kidneys, liver, and spleen) [16], and muscle mass decreases with aging [17], these changes appear slowly and over a long period. On the contrary, the thymus, which reaches its maximal size and T cell output during early postnatal life, exhibits early thymic involution, a phenomenon that becomes even more prominent with advancing age [12, 13, 18–21]. Although the size of the human thymus seems to remain unchanged throughout life under normal conditions [22], in other vertebrates, it declines during aging [23]. Nevertheless, in almost all vertebrates having a thymus, thymic cellularity is progressively decreased and replaced by adipose tissue over time, resulting in perturbation of the normal tissue architecture [14, 21, 24, 25] (Figure 1). Since T cell production is proportional to thymic epithelial tissue mass [26], thymic involution results in significant loss of its capability for *de novo* generation of immunocompetent T cells (Figure 1). The net outcome is a decline in frequency and function of naïve T cells, leading to a restricted T cell repertoire in the periphery [12, 27]. These changes may be at least in part responsible for the enhanced susceptibility and severity of infections, poor responsiveness to vaccination, and increased propensity for cancers and autoimmune diseases in the elderly [12, 28–32].

**Figure 2:**
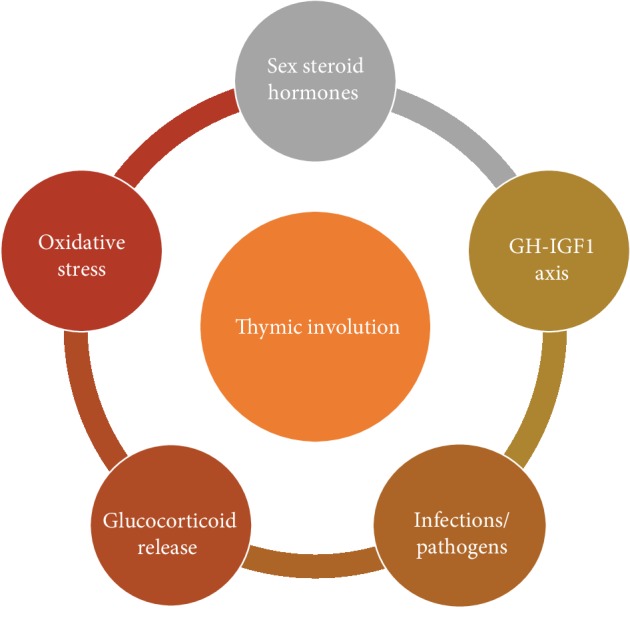
Proposed mechanisms involved in the pathophysiology of thymic involution. It must be highlighted that none of them can thoroughly explain this well-conserved biological phenomenon.

Even though age-associated thymic regression represents one of the most recognizable features of the aging immune system, the underlying mechanisms are not well understood [33]. Several candidates have been proposed, suggesting that thymic regression involves the interplay of various and different mechanisms (Figure 2); interestingly, there are lines of evidence that in this complex process, the thymic stroma and especially the TECS are the most sensitive compartment [12, 23, 27, 34]. A number of studies reported that sex steroid hormones, and especially androgens, contribute to age-associated thymic involution [12, 23, 27, 35] (Figure 2). This notion was based on the observations (a) that thymic involution, although beginning in early postnatal life, is more pronounced with the onset of puberty when sex steroid levels increase and (b) that high doses of sex steroid administration cause degeneration of the thymus (reviewed in [23]). Moreover, androgen impairment or ablation reduces thymic atrophy, while castration induces robust regeneration of the atrophied thymus; in the latter case, however, although androgen reduction is permanent, thymus rebound is only a transient response [12, 23, 27]. This observation, combined with the finding that thymus involution begins soon after birth, supports the notion that although the thymus is extremely sensitive to sex steroids, these hormones are not the predominant factors that induce thymus involution [23]. In addition, it cannot be explained why the thymus involutes at a faster rate than other tissues.

Numerous studies have also implicated the growth hormone- (GH-) insulin-like growth factor- (IGF-) I axis in thymus regression [23, 36, 37] (Figure 2). Both hormones promote thymic growth, and lately, GH has been used as an alternative strategy to rejuvenate the thymus in certain immunodeficiency disorders associated with thymic atrophy [38]. GH and IGF-I have been also considered as regulators of age-associated thymic involution, since GH production declines with age. However, the effects of hormone treatment on thymus size in older mice are limited, implying that there are other factors that prevent thymic atrophy [36].

The phenomenon of infection-induced inflammation and consequently thymus regression has also been reported; in human studies as well as in animal experimental models, infections with pathogens led to thymic atrophy, although the underlying mechanisms have not been extensively studied [35] (Figure 2). In addition, other periodical events such as glucocorticoid release as a response to different physiological stressors may also reduce the size of the thymus [21] (Figure 2). These theories, however, cannot explain the premature irreversible age-related thymic involution or why the thymus involutes at a faster rate than other organs.

Lately, a new player suggested to be involved in accelerated thymus involution and dysfunction with age is oxidative stress (Figure 2). Notwithstanding that the generation of reactive oxygen metabolites is an integral feature of normal cellular metabolism, the accumulation of such genotoxic and proteotoxic oxygen-derived by-products seems to exert detrimental effects on thymic tissue. Contrariwise, genetic or biochemical enhancement of antioxidant activity has been proven to ameliorate thymic atrophy [39].

Similarly, oxidative damage is also a well-documented inducer of cellular senescence, a state of permanent cell cycle arrest that allows cells to deal with various intrinsic and extrinsic stressors, in order to preserve cellular and, in a broader perspective, organismal homeostasis [40–45]. Except for cell cycle arrest, senescent cells may acquire numerous cellular and subcellular features, such as apoptosis resistance, increased galactosidase (SA-b-gal) activity, aberrant autophagic flow, lipofuscin accumulation, metabolic deregulation, and a complex prooxidant secretome (a feature termed senescence messaging secretome (SMS) or senescence associated secretory phenotype (SASP)) [40, 42, 43, 45–47]. Interestingly, the temporal activation of this adaptive stress response mechanism during embryonic development or normal adult life is linked with beneficial properties, whereas chronic senescence due to persisting stress and/or insufficient clearance of senescent cells by the immune system seems to exert detrimental effects [40, 43, 45–51]. In the latter case, the accumulation of senescent cells maintains an inflammatory milieu (*inflamm-aging*) that causes tissue remodeling, affects the regenerative potential and proper function of tissues/organs due to exhaustion of progenitor and stem cells, and, ultimately, promotes aging and age-related pathologies [45, 52–61].

Considering that (1^st^) oxidative stress has been linked to both the induction of cellular senescence and thymic involution and (2^nd^) aging is characterized by accumulation of senescent cells as well as a decline in thymus function, it is not unreasonable to assume that cellular senescence may exert a critical role in the induction of thymic involution, with oxidative stress being the common denominator. Indeed, recent evidence, from human and animal studies, supports this notion [62, 63]. The scope of this review is to summarize data regarding the role of oxidative stress in both thymic involution and cellular senescence. For a more comprehensive approach, we first present an overview regarding *in vivo* oxidative stress, antioxidant mechanisms, and the related aging theory.

## 2. Oxidative Stress and Related Aging Theory

Higher eukaryotic aerobic organisms depend on oxygen for efficient production of energy in mitochondria, yet oxygen is a toxic mutagenic gas jeopardizing cellular and, thus, organismal homeostasis [64]. This “oxygen paradox” derives from the chemical properties of molecular oxygen, which predispose to the generation of free radicals and other reactive oxygen species (ROS) in aerobes [64]. In particular, the diatomic oxygen molecule (O_2_) is a free radical: it contains two unpaired electrons with parallel spins located in separate orbitals [65]. Therefore, if O_2_ attempts to oxidize another atom or molecule by accepting a pair of electrons, both electrons must have the same spin. This “spin restriction” limits O_2_ to reduction reactions that involve single electron transfers and explains why O_2_ is itself a weak oxidizing agent [66]. However, some of its metabolites are potent oxidants that can directly oxidize various substrates [67]. Luckily, most of the O_2_ (over 95%) received *via* breathing in humans is reduced directly to water in the mitochondrial electron transport chain (ETC), as it accepts four electrons and four protons (H^+^) in a reaction catalyzed by the enzyme cytochrome oxidase. ETC is coupled with oxidative phosphorylation to produce energy in adenosine triphosphate (ATP) form. Thus, the main function of oxygen in life is to serve as an electron acceptor for efficient energy production. Unfortunately, this concerted tetravalent reduction of O_2_ by the mitochondrial ETC, to produce water, is not without “collateral damage.” Accidental electron leakage from components of the mitochondrial respiratory chain may occur; therefore, O_2_ may undergo univalent reduction which generates ROS, such as the superoxide anion (O_2_^·-^) and the hydrogen peroxide (H_2_O_2_) which are moderately reactive, and the extremely reactive and consequently short-lived hydroxyl radical (^·^OH) [64, 67]. *In vitro*, mitochondrial respiration is responsible for up to 2% of the consumed O_2_ into O_2_^·-^, whereas *in vivo*, the rate seems to be much lower [68, 69]. Excessive amounts of ROS are also produced by stimulated phagocytes via the enzyme nicotinamide adenine dinucleotide phosphate (NADPH) oxidase. O_2_^·-^ and oxidants derived from phagocytic NADPH oxidase are crucial components of the immune defense system [67]. Except for mitochondria and phagocytic cells, ROS are also produced by endoplasmic reticulum, plasma membranes, lysosomes, and peroxisomes [67]. Moreover, in addition to endogenous release due to cellular metabolism, oxygen-derived reactive by-products may arise from interactions with exogenous sources such as environmental pollution, ionizing or solar radiation, xenobiotic metals, drugs, or cigarette smoke [64, 70].

Cellular macromolecules, such as nucleic acids, proteins, lipids, and carbohydrates, are vulnerable to the toxic effect of these oxygen-derived intermediates. Obviously, aerobes are not defenseless, as they utilize a series of highly effective mechanisms in an attempt to be protected against oxidative damage [64]. The major burden of antioxidant defense is shouldered by scavenger enzymes, synthesized by all known aerobic organisms, whose role is to metabolize and reduce ROS to less reactive or totally innocuous molecules [71, 72]. In this regard, superoxide dismutases (SOD) catalyze the dismutation of O_2_^·-^ into H_2_O_2_ and O_2_, while H_2_O_2_ is mainly eliminated by an enzymatic network including catalases (CAT), peroxiredoxins (PRX), and glutathione peroxidases (GPX) [64, 73]. Notably, in the presence of unshielded redox-active iron, often referred as “labile iron,” H_2_O_2_ can also be reduced nonenzymatically to form the highly toxic ^·^OH via the Fenton reaction [74–77]. Aerobic organisms use additionally lipid- and water-soluble antioxidant “sacrificial” compounds such as vitamins C and E, carotenoids, and glutathione (GSH) that are preferentially oxidized to preserve more important biomolecules, as well as defense mechanisms that remove or repair oxidized cellular components [64].

Apparently, in physiological conditions, the continuous generation and elimination of ROS are finely regulated, resulting in a dynamic steady-state ROS level, which varies among cell types and intracellular compartments. However, under certain circumstances, this oxidant-antioxidant balance can be disrupted in favor of the oxidants, resulting in what is termed a state of oxidative stress. The concept of oxidative stress was introduced by Helmut Sies in 1985 [78] and it is currently defined as “an imbalance between the oxidants and antioxidants in favor of the oxidants, leading to a disruption of the redox signaling and control and/or molecular damage” [79]. The consequences of oxidative stress are related to the dosage, type, duration, and site of ROS formation. Slight variations in the cellular redox balance initiate transduction of signals (called “redox signaling”), mainly through reversible modification of specific target proteins, and play a plethora of physiological roles [70]. A noteworthy feature of ROS is that they can initiate adaptive responses which allow cells to counteract with shifts in the level of oxidative stress and restore cellular homeostasis [70]. Mild exposure to oxidants induce transient cell cycle arrest and adaptive alterations in gene expression [80]. It is well established that many of the components of the antioxidant defense mechanisms are activated in response to ROS [80]. Nevertheless, at higher levels of ROS, oxidative damage accumulates and cells may enter a permanent arrest of the cell cycle (senescence) or may commit “suicide” by activating apoptotic death programs, in order to protect surrounding healthy tissue from further damage. Finally, under extreme conditions, necrotic death with disposal of cell corpses occurs, leading to inflammatory immune responses and damage in adjacent cells [80].

Oxidative stress has been implicated in numerous pathologies including aging [64, 81–84]. Aging is an inevitable biological process, characterized by a functional decline that leads to increased morbidity and mortality. Among the theories proposed to explain its molecular base, the “free radical theory of aging” has attracted great interest and has accumulated substantial experimental support [85, 86]. Although there are studies questioning this theory [87, 88], a consensus exists that ROS produced even during normal aerobic metabolism cause cumulative oxidative damage which challenges the integrity of the genome and proteome, thus compromising cellular homeostasis and contributing to organismal aging.

Mitochondria seem to have a central role regarding oxidative stress as they may be endogenous sources and simultaneously targets of ROS [89–92]. In fact, failure of mitochondrial quality control systems, particularly the disruption of mitochondrial genome integrity, is clearly associated with aging phenotypes [93]. Of note, oxidative stress is the most common mechanism of damage as far as mitochondrial DNA is concerned [94]. In this respect, it has been recently demonstrated that transient mtDNA double-strand breaks (DSBs) cause an accelerated aging phenotype, preferentially affecting proliferating tissues [95]. Fortunately, the integrity of the mitochondrial genome is protected by a three-level defense system, including (a) the structure of mitochondrial DNA (mtDNA), (b) the DNA repair mechanisms functioning within mitochondria, and (c) mitochondrial dynamic processes (i.e., fusion, fission, and mitophagy) that “recycle” damaged mtDNA [96–98]. Notably, well-known manipulations that extend lifespan (e.g., caloric restriction and exercise) have been suggested to maintain mitochondrial homeostasis and modulate mitochondrial oxidative activity [99–102]. Interestingly, the classic mitochondrial free radical theory of ageing, according to which mtDNA mutations cause genotoxic oxidative stress and this in turn creates more mutations, has currently been challenged [93]. New hypotheses of how age-associated mitochondrial dysfunction may lead to aging have emerged. These novel insights highlight the role of ROS as signaling molecules and focus on their role in mediating stress responses, such as cellular senescence [93, 103, 104].

## 3. Oxidative Stress in Age-Related Thymic Involution

Oxidative stress has been implicated in the aging process as well as in several age-related diseases [64, 81, 82, 84]. Lately, a number of animal studies associated increased oxidant status with the reduction in thymus size, suggesting that oxidative stress may contribute to the thymic involution observed during aging. In these studies, subjects were exposed to various stimuli that are known to induce the production of ROS *in vivo* and a number of biomarkers were used to assess the extent of oxidative stress [39, 105–111]. These include biomarkers measuring ROS-induced modifications in essential biomolecules, activity of ROS-generated enzymes, or ROS-detoxifying enzymes. Notably, administration of compounds with antioxidant and free radical-scavenging activity significantly decreased oxidative damage and restored the size of the thymus [105, 107, 109, 112].

Consistent with these findings, mice exposed repetitively to ozone which is a potent oxidizing agent, exhibited accelerated thymic involution [113]. In this study, a decline in thymus weight index was observed, which was proportional to the dose of inhaled ozone [113]. Recent evidence in mice models showed also a link between ROS, mtDNA damage, and thymic shrinkage [95]. In this study, a mouse model that ubiquitously induces mtDNA double-strand breaks exhibited thymic shrinkage along with accelerated apoptosis, senescence, and adipose tissue differentiation, mimicking age-related thymic involution. This phenotype was also accompanied by increased production of H_2_O_2_ and activation of cell cycle arrest proteins which were reverted by treatment with MitoQ (a mitochondrial-targeted antioxidant) and n-acetylcysteine (an antioxidant and free radical scavenger) [95]. Similarly, in an aging rat model employed by D-galactose treatment, both thymic atrophy and elevated oxidative stress levels were reported [114]. Again, saponin supplementation (an antioxidant derivative from the plant *Aralia taibaiensis*) attenuated oxidative stress-induced aging traits, including thymus shrinkage. Notably, both the FOXO3a (Forkhead box O3) pathway and the Nrf2 (nuclear factor erythroid 2-related factor 2) pathway were involved in the protective process [114]. Moreover, a mitochondria-targeted antioxidant has been shown to delay thymic atrophy in both normal and senescence-prone rats [115]. OXYS rats which are fast senescence-prone rats show shortened lifespan, early development of age-associated phenotypes including premature thymic involution, and decreased function of T cell-dependent immunity. Moreover, they often exhibit higher levels of oxidative damage. The mitochondria-targeted antioxidant SkQ1 (plastoquinonyl decyltriphenyl phosphonium) delayed age-related processes as well as age-dependent thymic involution in both normal rats and OXYS rats.

**Figure 3:**
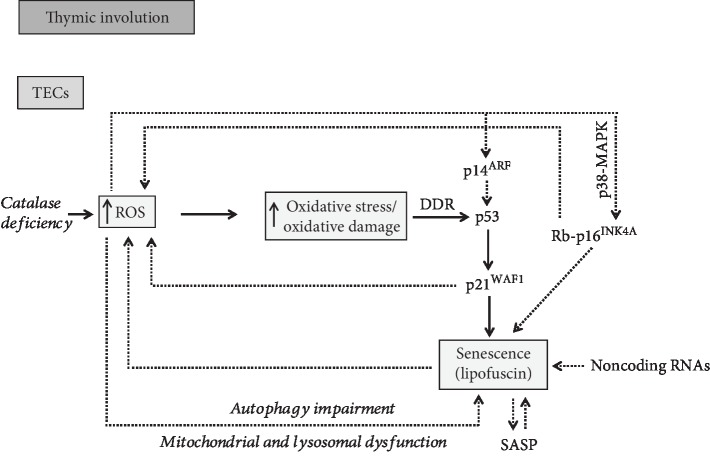
Overview of putative mechanisms involved in oxidative stress-induced cellular senescence in age-related thymic involution.

The above studies provide interesting, although circumstantial, information, for the link between oxidative stress and age-related thymic atrophy. Direct experimental evidence for their cause-effect relationship has been reported recently in mice models [39]. This study shows that in contrast to lymphoid cells, stromal cortical and medullary TECs are relatively deficient in the H_2_O_2_-metabolizing enzyme CAT. Therefore, stromal cells exhibit increased ROS levels and elevated DNA damage (Figure 3). Experiments that biochemically enhanced antioxidant activity by dietary supplementation of antioxidants (N-acetylcysteine or L-ascorbate) showed significant inhibition of thymic atrophy. Similar results were observed in transgenic mice ubiquitously expressing *Cat* in mitochondria, providing strong evidence that thymic atrophy is impacted by the redox state, and furthermore, it is ameliorated when ROS formation is prevented in mitochondria, the principal site of ROS generation [39]. Regarding the role of mitochondrial ROS in mediating cellular or tissue damage, the targeted overexpression of *Cat* in mitochondria significantly attenuates oxidative damage and furthermore increases lifespan and healthspan in mice [116, 117].

A question arising is how *Cat* deficiency in TECs induces thymic regression, since this enzyme metabolizes high levels of H_2_O_2_. In particular, it is predominantly colocalized with H_2_O_2_-generating oxidases in peroxisomes, where it functions against the toxic effects of generated peroxides and acts when H_2_O_2_ reaches high intracellular concentrations [118–120]. However, in lower and more physiological concentrations, the main scavengers of H_2_O_2_ are GPX and PRX, which exert a wider subcellular activity [121]. Interestingly, the overall outcomes of *Cat* knockout are rather limited, compared to other H_2_O_2_-metabolizing enzymes [73]. In this regard, homozygous *Cat* knockout mice develop normally and are apparently healthy, although they display tissue-dependent oxidant-mediated injury, especially under conditions of oxidative stress [122].

The fact that thymic involution begins soon after birth could be explained, at least in part, by considering that the thymus which supports thymopoiesis is an extremely anabolic (lymphoid) environment [39, 123]. Indeed, thymopoiesis occurs more robustly during late embryonic and perinatal period, and it has been suggested that it is likely responsible for the robust decrease in the numbers and/or activity of medullary TEC stem cells which occurs soon after birth [123]. This effect on TECs can be explained because thymopoiesis involves both high rate of proliferation and massive apoptosis of thymocytes (thymocytes undergo both positive and negative selection within the thymus) resulting in the accumulation of potentially damaging factors such as ROS which may affect TECs [123]. In this context of robust thymopoiesis, an inherent deficiency of TECs in CAT may render these cells more vulnerable to ROS and could contribute to early thymic atrophy (Figure 1), thereby providing evidence to explain, at least in part, why the thymus is affected more rapidly in comparison to other organs [39]. Relevant to the above analysis is our previous study, in which we showed for the first time in human thymuses that TECs but not lymphoid cells exhibit oxidative DNA damage in samples from aged individuals [63]. These findings provide *in situ* evidence the TECs but not lymphoid cells are sensitive to ROS [63].

Yet, despite their effect in thymic atrophy, preliminary data address the possibility that ROS are required for specialized functions of stromal cells in mice and thus play physiological roles. In particular, ROS produced by thymic stromal cells seem to be necessary for autophagy which is essential for negative selection and thus for the prevention of autoimmune diseases [124]. Probably, ROS play a dual role in the thymus: they may be involved in the establishment of central T cell tolerance in the young steady-state thymus, while the resulting accumulated oxidative damage can eventually lead to thymic atrophy during aging.

Among several different types of ROS, H_2_O_2_ has emerged as a central hub in both oxidant-mediated damage and redox-signaling networks [125] although the underlying molecular mechanisms remain unclear. Lately, it has been reported that “labile iron,” a small cellular fraction of poorly characterized unshielded redox-active iron, determines diverse H_2_O_2_-mediated effects [126–129]. It seems that the labile iron pool plays a major role not only in H_2_O_2_-induced toxicity but also in specific redox signaling pathways [75, 76]. Of note, iron accumulates in aged tissues and the dysregulation of iron homeostasis has been implicated in the development of aging phenotypes [83, 130–135]. Nevertheless, the potential contribution of iron in thymic involution has not yet been examined.

## 4. Cellular Senescence in Age-Related Thymic Involution

Oxidative damage is a well-known trigger of cellular senescence. Internal elevated ROS levels can directly damage cellular components through the oxidation of biomolecules or can act as second messengers to modulate specific signaling cascades [136]. The direct damaging of DNA induces the DNA damage response (DDR) pathway that in turn activates p53 and its downstream effector p21^WAF1^, leading to one of the following alternative outcomes in an attempt to repair these lesions: reversible cell cycle arrest, senescence, or apoptosis [137] (Figure 3). Additionally, p21^WAF1^ activation promotes a steady increase in ROS generation that further fuels DNA damage. This positive feedback seems to be both necessary and sufficient to maintain cell cycle arrest during establishment of irreversible senescence [138]. In addition, senescence can be induced upon DNA damage via the Rb- (retinoblastoma protein-) p16^INK4A^ axis (Figure 3). Of note, it has been suggested that while p53 and p21^WAF1^ act to initiate the senescence response, p16^INK4A^ functions in the maintenance of this state. Interestingly, p16^INK4A^ can be activated in response to oxidative stress through the p38-MAPK (mitogen-activated protein kinase) pathway [139] (Figure 3). p38 belongs to the stress-activated protein kinase family (SAPK) that responds to a variety of physiologic stresses, including oxidative stress [140]. Moreover, the p16^INK4a^/Rb pathway is implicated in a ROS-dependent positive feedback loop, which reinforces the irreversible cell cycle arrest in senescent cells [141]. Moreover, p14^ARF^, the alternate reading frame protein product of the INK4A (inhibitor of cyclin-dependent kinase 4)/ARF (alternate reading frame) locus, has also been shown to play an important role in the long-term stabilization of p53 following DNA damage. As previously shown, this protein acts as a sensor of oxidative stress [142] and plays an important role, under circumstances, in mediating senescence. Importantly, p14^ARF^ also interplays with the DNA damage response pathway, as previously shown, in order to exert p53-independent antitumor functions [143].

Of great importance, lipofuscin, a nondegradable material that accumulates in the cytoplasm of senescent cells, mediates the release of excessive amounts of ROS and impairs the ubiquitin proteasome pathway (UPP), the principal mechanism for catabolism of dysfunctional proteins in the mammalian cytosol and nucleus. [144, 145]. In this manner, a damaging feedback loop that fuels oxidative stress and promotes aging occurs.

Given that oxidative stress can induce senescence and senescent cells accumulate in tissues as a consequence of aging [57], we investigated whether senescence is present during age-related thymic involution in human archival (formalin fixed and paraffin embedded) material [63]. For this purpose, we used a novel, biotinylated Sudan Black B analogue termed GL13 (SenTraGor™) that specifically reacts with lipofuscin, a hallmark of senescent cells [41, 44, 63, 146, 147]. Indeed, a number of TECs were detected mainly within the medulla and especially in the vicinity of Hassall's corpuscles that exhibited dense cytoplasmic GL13 positivity in the majority of aged samples whereas staining in sections from infants was negative [63] (Figure 3). The GL13-positive TECs showed simultaneously nuclear p21^WAF1^ immunopositivity in double-staining experiments, further confirming their senescent phenotype [63] (Figure 3). As previously shown, these cells undergo cell cycle arrest (p16^INK4A^ and p21^WAF1^ positive), while proliferation and apoptotic markers showed low expression or were negative [3, 148]. Interestingly, medullary TECs exhibited also 8-oxoG and *γ*-H2AX (H2A histone family member X phosphorylated on serine 139) immunoreactivity, suggesting that a relationship between cellular senescence and oxidative DNA damage exists in the aged human thymus [63]. Similar findings in mouse models demonstrated increased *β*-galactosidase activity and strong *γ*-H2AX immunopositivity in TECs [62]. In addition, it was lately shown that in aged mice, the subcapsular space of the thymus exhibited high density of pigmented bodies [149], autofluorescent inclusions which change with age, and represent hallmarks of aging [150]. Putative insights into oxidative stress-driven senescence during thymic involution will be next discussed, based on the current state of knowledge.

Besides senescence, perturbed autophagy has also been associated with the aging process; thus, a link between autophagy and thymic involution seems possible. The autophagic process appears to play significant roles in the thymic histophysiology [8, 9, 151]. Autophagy supports central CD4-positive T cell tolerance through facilitating the presentation of endogenous self-antigens by medullary TEC [151]. The immunohistochemical expression of lysosome-associated membrane proteins 1 and 2 (LAMP-1 and LAMP-2) was investigated in thymuses [152, 153] since LAMP-2 participates in chaperone-mediated autophagy [154] and evidence was provided for differential expression of LAMP-1 and LAMP-2 in thymic involution [152]. Indeed, in acute (infection induced) involution, LAMP-1 was observed mainly in medullary TEC, in single macrophages and thymocytes [152]. Hassall's corpuscles were immunostained less intensely as compared with control tissues, and the quantitative study revealed a significantly increased LAMP-2 immunoexpression compared with LAMP-1 [152]. In chronic (senile) involution, LAMPs were observed with very slight immunoreactivity [152]. The increased expression of LAMPs, and mainly of LAMP-2, in the TEC of thymuses with acute (infection induced) involution might indicate acute cell injury requiring autophagic degradation of damaged thymic structures, and the reduced expression of LAMPs in the age-involuted (senile) thymus might indicate thymic morphological reorganization and functional dysregulation [152]. The immunohistochemical expression of the autophagy regulator microtubule-associated protein 1A/1B-light chain 3 (LC3) was greatly reduced in the thymus of 12- and 24-month-old mice while an ultrastructural study revealed that the number of autophagic structure/vacuoles in total TEC decreases with age [155]. It was suggested that the age-related decrease in autophagic structures in TEC may reduce the immunocompetent T cell pool in aged mice [155].

An interplay between autophagy and ROS in aging has also been reported. Studies in invertebrate model organisms show that autophagy defects cause sensitivity to oxidative stress and decrease longevity, while overexpression of autophagy genes extends the average lifespan and enhances resistance to oxidative stress, indicating the fundamental role of autophagy in oxidative stress-induced organismal aging [156]. A link between impaired autophagy and oxidative stress in aging and age-related pathologies is anticipated. Aerobes are exposed to a permanent flux of ROS which increases during aging, resulting in the accumulation of oxidized or dysfunctional cellular structures [83, 150, 157, 158]. In turn, autophagy declines with age [159, 160], rendering cells unable to remove damaged components formed as a consequence of metabolic stress. Thus, oxidative damage is cumulative during the lifespan, causing gradual decrease of cellular functions and resulting in aging and age-related diseases [83, 150].

Recently, evidence on how impaired autophagy promotes oxidative damage-induced senescence in murine fibroblasts and human fetal lung fibroblasts was reported supporting the inverse correlation between these two cellular processes [161] (Figure 3). Furthermore, mitochondrial dysfunction has emerged as a causal player of cellular senescence [104, 162–164]. It seems that mitochondrial dysfunction and increased endogenous ROS formation are the common denominator for both cellular senescence and aging [138, 161, 163, 165, 166]. Mitochondrial dysfunction temporally precedes lysosomal dysfunctions and may be the initiating event, while lysosomal dysfunction, although occurring later, is more directly responsible for the impaired autophagic activity and senescence development [161] (Figure 3). “The mitochondrial-lysosomal axis theory of aging” supports that the driving force for organismal aging relies in the dysfunction and mutual effect of these two organelles [167, 168]. Lately, studies gained insight into the redox signaling pathways regulating autophagy and senescence. The AMP-activated protein kinase (AMPK) is known to activate autophagy and prevent senescence. Han et al. determined that senescence induced in fibroblasts and umbilical vein endothelial cells by H_2_O_2_ treatment was accompanied with inactivation of the AMPK pathway, autophagic dysfunction, and decreased NAD+ (nicotinamide adenine dinucleotide) levels which is a known feature of aged organisms [169]. Importantly, pharmacological activation of AMPK prevented senescence, improved the impaired autophagic flux, and restored NAD+ synthesis, thus suggesting that AMPK targets multiple pathways in cells, to collaboratively prevent oxidative stress-induced senescence. Similarly, impaired autophagic processes and AMPK dysfunction-induced oxidative stress-related senescence comprise an auditory cellular premature senescence model [170]. Moreover, an inverse relationship, where genetic impairment of autophagy promotes premature senescence, was reported in primary human fibroblasts in a ROS- and p53-dependent mechanism [171]. In this study, suppression of autophagy stimulates senescence through activation of the p53 tumor suppressor pathway due to ROS overproduction. Presumably, in this case, an increased mitochondrial ROS generation occurs as a consequence of the reduced turnover of dysfunctional mitochondria due to autophagy inhibition. Furthermore, the chemical block of p53 or ROS scavengers delays the premature senescence by improving the autophagic function, suggesting the existence of a feedback mechanism between ROS-induced p53 activation and autophagy. Overall, these data suggest that p53 is an effector of autophagy-induced senescence triggered via ROS signaling.

## 5. Conclusions

Thymic involution is associated with a progressive decline in the output of naïve T cells and a restricted T cell repertoire in the periphery, resulting in impaired immune responses to new pathogens or vaccines, increased incidence of specific cancers, and certain autoimmune disorders in the elderly subjects [12]. Although it is unclear what constitutes the driving forces resulting in early thymic involution [12], evidence supports that oxidative damage in the stromal compartment is one of the potential candidates [39]. We recently showed *in situ* the correlation of cellular senescence with thymic involution in human clinical settings [63] (Figure 3). In addition, we provided evidence that senescence during human thymic involution is related to increased oxidative DNA damage [63], a finding that is in line with previous observations showing that stromal mouse thymic cells, but not lymphoid cells, exhibit an inherent deficiency in the H_2_O_2_-reducing enzyme CAT [39] (Figure 3).

Based on these data, we suggest that oxidative stress may be one of the factors inducing senescence in stromal thymic cells including TECs. Of note, although TECs are of epithelial origin, they exhibit unique features such as a low turnover rate and function that may resemble the stromal compartment in other organs. Therefore, induction of senescence in TECs due to elevated oxidative stress levels during thymic involution is in line with our previous proposed concept that “stromal” cells tend to respond to stress by entering senescence [172]. In contrast, T lymphocytes which are present in the thymus transiently seem to undergo apoptosis. Taken together, these findings provide new insights in understanding normal thymus histophysiology and how cellular senescence is implicated in age-related processes such as thymic involution. The discovery of senolytic drugs that can reverse senescence could raise some interesting issues [173, 174]. Clearance of senescent cells from the thymus may have beneficial effects considering its premature involution. Nevertheless, the detrimental effects of thymic involution have been questioned. Initially, it seems like a disadvantage for the organism, as it increases the risk of infection and the incidence of cancers, due to the restriction in T cell repertoire. However, it might be an evolutionary advantage, as the above detriments are offset by a reduced risk of autoimmune diseases [175]. Thus, thymic involution may be regarded as an adaptation of the organism to aging. Although this theory requires experimental support, it should be taken into consideration as it raises questions for the putative beneficial effect of restoring the thymic activity in elderly. Finally, the exact role of cellular senescence during thymic tumorigenesis (thymic epithelial tumors) [176, 177], given its bimodal mode of action in cancer, can now be further examined with the use of the novel GL13 compound in clinical material. In this context, it would also be interesting to investigate the implication of senescence in the response to therapy and regression of thymic tumors in order to improve the outcome of patients.

## Abbreviations

AMPK: Adenosine monophosphate-activated protein kinase
ARF: Alternate reading frame
ATP: Adenosine triphosphate
CAT: Catalase
DCs: Dendritic cells
GL13: Sudan Black B analogue
DDR: DNA damage response
DSBs: Double-strand breaks
ETC: Electron transport chain
FOXO3: Forkhead box O3
GSH: Glutathione
GPX: Glutathione peroxidase
H_2_O_2_: Hydrogen peroxide
INK4A: Inhibitor of cyclin-dependent kinase 4
LAMP-1: Lysosome-associated membrane protein 1
LAMP-2: Lysosome-associated membrane protein 2
LC3: Microtubule-associated protein 1A/1B-light chain 3
MAPK: Mitogen-activated protein kinase
mtDNA: Mitochondrial DNA
NADPH oxidase: Nicotinamide adenine dinucleotide phosphate oxidase
NAD: Nicotinamide adenine dinucleotide
Nrf2: Nuclear factor erythroid 2-related factor
OH: Hydroxyl radical
PRX: Peroxiredoxin
ROS: Reactive oxygen species
Rb: Retinoblastoma protein
SASP: Senescence associated secretory phenotype
SMS: Senescence messaging secretome
SAPK: Stress-activated protein kinase family
O_2_: Superoxide anion
SOD: Superoxide dismutase
TECs: Thymic epithelial cells
UPP: Ubiquitin proteasome pathway
8-oxoG: 8-Oxoguanine
*γ*-H2AX: H2A histone family member X phosphorylated on serine 139.
